# *EGFR* Polymorphism and Survival of NSCLC Patients Treated with TKIs: A Systematic Review and Meta-Analysis

**DOI:** 10.1155/2020/1973241

**Published:** 2020-03-18

**Authors:** Vladimir Jurisic, Vladimir Vukovic, Jasmina Obradovic, Lyudmila F. Gulyaeva, Nikolay E. Kushlinskii, Nataša Djordjević

**Affiliations:** ^1^Faculty of Medical Sciences, University of Kragujevac, Kragujevac, Serbia; ^2^Institute for Biomedicine, Eurac Research, Affiliated Institute of the University of Lübeck, Bolzano, Italy; ^3^Institute for Information Technologies, Department of Sciences, University of Kragujevac, Kragujevac, Serbia; ^4^University of Medicine, Novo Sibirsk, Russia; ^5^N. N. Blokhin National Medical Research Center of Oncology, Moscow, Russia; ^6^Department of Pharmacology and Toxicology, Faculty of Medical Sciences, University of Kragujevac, Kragujevac, Serbia

## Abstract

Tyrosine kinase inhibitor- (TKI-) based therapy revolutionized the overall survival and the quality of life in non-small-cell lung cancer (NSCLC) patients that have epidermal growth factor receptor (*EGFR*) mutations. However, *EGFR* is a highly polymorphic and mutation-prone gene, with over 1200 single nucleotide polymorphisms (SNPs). Since the role of *EFGR* polymorphism on the treatment outcome is still a matter of debate, this research analyzed the available literature data, according to the PRISMA guidelines for meta-analyses. Research includes PubMed, Scopus, ISI Web of Science, and 14 of genome-wide association studies (GWAS) electronic databases in order to provide quantitative assessment of the association between ten investigated *EGFR* SNPs and the survival of NSCLC patients. The pooled HR and their 95% CI for OS and PFS for different *EGFR* polymorphisms using a random or fixed effect model based on the calculated heterogeneity between the studies was applied. The longest and the shortest median OSs were reported for the homozygous wild genotype and a variant allele carriers for rs712829 (-216G>T), respectively. Quantitative synthesis in our study shows that out of ten investigated *EGFR* SNPs (rs11543848, rs11568315, rs11977388, rs2075102, rs2227983, rs2293347, rs4947492, rs712829, rs712830, and rs7809028), only four, namely, rs712829 (-216G>T), rs11568315 (CA repeat), rs2293347 (D994D), and rs4947492, have been reported to affect the outcome of TKI-based NSCLC treatment. Of these, only -216G>T and variable CA repeat polymorphisms have been confirmed by meta-analysis of available data to significantly affect OS and PFS in gefitinib- or erlotinib-treated NSCLC patients.

## 1. Introduction

For the past several decades, lung cancer remains one of the major causes of mortality worldwide [1–3]. According to the World Health Organization, it is the most commonly diagnosed cancer and the leading cause of cancer death, with over 2 million of new cases and more than 1.7 million deaths in 2018 [4, 5]. Of those, over 85% is due to non-small-cell lung cancer (NSCLC), which exhibits better prognosis than its complement, i.e., small cell lung cancer [1], yet displays low long-term survival and reduced quality of life [6, 7]. Although cigarette smoking represents the primary risk factor for NSCLC development [8], numerous investigations confirmed that genetics plays one of the leading roles in the process [9–11]. Gene variations that have been identified as conferring higher risk of NSCLC could be either germline or somatic, with some of the most common lung cancer-related driver mutations linked to epidermal growth factor receptor gene (*EGFR*) [12].

*EGFR* is a transmembrane tyrosine kinase receptor that, upon activation, becomes a transducer of signals for cell proliferation [13]. *EGFR* overexpression, often due to genetic alterations, has been firmly and consistently associated with carcinogenesis [13–15], and *EGFR* itself recognized as a potential target of an important therapeutic approach to cancer. Namely, it has been observed that drugs that inhibit tyrosine kinases, enzymes important for tumor cell proliferation, growth, and metastasis, display target-specific antitumor activity against different types of malignancies, including lung, breast, colorectal, and prostate cancer [16]. Since the discovery of gefitinib, the first tyrosine kinase inhibitor (TKI) aimed *EGFR* [17], several similar drugs have been approved for the treatment of NSCLC, including erlotinib [18, 19]. Compared with chemotherapy as a former treatment of choice, TKI-based therapy revolutionized the overall survival and the quality of life of NSCLC patients, especially if they are carriers of the *EGFR* driver mutations [20–23]. Still, for the majority of patients, the prognosis of NSCLC remains unfavorable, mainly as a consequence of either intrinsic or acquired resistance to TKI. While acquired resistance develops during the treatment, mostly due to occurrence of secondary *EGFR* mutations, intrinsic resistance usually implies the presence of inherited variations, including *EGFR* single-nucleotide polymorphisms (SNPs) [24–27].

*EGFR* is highly polymorphic and mutation-prone gene, with over 1200 SNPs [28] and over 2700 mutations [29] described so far. *EGFR* mutations have been extensively studied in relation to NSCLC, and some of them, including alterations in the tyrosine kinase domain, were clearly associated with better response to TKI-based therapy [30]. Yet, the role of *EFGR* polymorphism on the treatment outcome is still a matter of debate, as published research studies offer inconsistent results [31, 32], and available meta-analyses lack the comprehensiveness in terms of included SNPs [25, 33]. Therefore, the aim of our study was to review and analyze the available literature on TKI-based therapy, in order to provide quantitative assessment of the association between *EGFR* polymorphism and the survival of NSCLC patients.

## 2. Methods

### 2.1. Literature Search and Study Selection

To identify the studies on the association between *EGFR* polymorphisms and the survival in NSCLC patients treated with TKI therapy, a systematic search of the available literature according to the Preferred Reporting Items for Systematic Reviews and Meta-Analyses (PRISMA) guidelines for meta-analyses and systematic reviews was performed [34]. Three electronic databases, namely, PubMed [35], Scopus [36], and ISI Web of Science [37], were thoroughly explored, with a search query consisting of a specific combination of subject headings and text words. For searching the PubMed database, the following combination of terms was used: ((“receptor, epidermal growth factor” [MeSH Terms] OR *EGFR* [All Fields]) AND (gene[tiab] OR “polymorphism, genetic”[MeSH Terms]) AND (“carcinoma, non-small-cell lung” [MeSH Terms] OR NSCLC [All Fields]) AND (((“drug therapy” [Subheading] OR treatment [All Fields] OR “erlotinib hydrochloride” [MeSH Terms] OR TKI OR “TK inhibitors” OR “tyrosine kinase inhibitors” OR “Tyrosine-kinase inhibitor”) AND response [All Fields]) OR Prognosis [MeSH]) AND (humans [MeSH])). Other two databases, i.e., Scopus and ISI Web of Science, were searched using the appropriately modified initial PubMed search query (details are available upon request). In addition, detailed search of several publically available databases of genome-wide association studies (GWAS) was carried out, including the GWAS Central [38], the Genetic Associations and Mechanisms in Oncology (GAME-ON) [39], the Human Genome Epidemiology (HuGE) Navigator [40], the National Human Genome Research Institute (NHGRI GWAS Catalog) [41], the database of Genotypes and Phenotypes (dbGaP) [42], the GWASdb [43], the Italian Genome-Wide Database (IGDB) [44], and the GRASP: Genome-Wide Repository of Associations between SNPs and Phenotypes [45]. Finally, we separately searched the bibliographies of eligible studies to look for additional studies. We considered studies published until February 09, 2018 and written in English, Italian, or Russian.

Studies were considered eligible if they assessed the association between the *EGFR* polymorphism in NSCLC patients treated with EGFR-TKI, and survival, expressed as the progression-free survival (PFS), time to progression (TTP), or the overall survival (OS). PFS was defined as the time from the first day of EGFR-TKI treatment until tumor progression or death from any cause while censoring the patients that were lost to follow-up [46]. Time from initiating the therapy until the disease progression as the event of interest was considered as the TTP [47]. Finally, OS was defined as the period from the first day of EGFR-TKI therapy to the date of death or final follow-up, whichever arrived first [47]. Hazard ratios (HRs) with corresponding 95% CIs were used to evaluate the quantitative aggregation of the survival for different genotypes of *EGFR*.

After all potentially eligible studies were collected, cross-linking of the studies from different electronic databases was performed in order to remove duplicates. Two reviewers (J.O. and V.V.) independently screened the titles and abstracts of the relevant articles, and any disagreement was resolved through discussion. Full texts of the potentially eligible studies were subsequently retrieved and assessed for final inclusion, according to the reported criteria. Namely, we only included studies conducted on patients with histopathologically confirmed NSCLC, who received EGFR-TKIs based therapy and where the measures of outcome were reported according to the *EGFR* genotype. On the other hand, reviews, meta-analyses, editorials, case reports, and studies conducted only on cell lines were excluded. When there were multiple publications on the same or overlapping study population, we only included the most comprehensive one.

### 2.2. Data Extraction and Quality Assessment

Two investigators (J.O. and V.V.) independently extracted data from each article into a database using a structured sheet. The following items were considered: (a) general: first author, year, country, study design, study period, and number of patients; (b) study subjects: median age, gender, ethnicity, percentage of smokers, clinical stage, and median follow-up period (months); (c) therapy: preparation therapy, main therapy line used; (d) *EGFR* genotype: genotyping platform used, variant location, the dbSNP-ID, and number of patients per genotype; (e) outcome: OS, TTP, and PFS with 95% CI.

The same investigators evaluated the methodological quality of included studies using the widely accepted Newcastle–Ottawa Quality Assessment Scale (NOS) for cohort studies [48] and the Jadad Scale for the randomized control trials (RCTs) [49]. The NOS for cohort studies evaluates three perspectives of the methodological quality: the selection of the study groups (four points); the comparability of the groups (two points); and the ascertainment of exposure or outcome of interest for cohort studies (three points) and assigns a total of maximum 9 points. The Jadad scale for reporting randomized controlled trials evaluates the risk of bias in three domains: randomization, double blinding, and description of withdrawals and dropouts with a final score from 0 to 5. Any disagreements between the reviewers were resolved through discussion or in consultation with other authors.

### 2.3. Statistical Analysis

Meta-analysis was conducted when at least two studies on the same genetic variant were available. We calculated the pooled HR and their 95% CI for OS and PFS for different *EGFR* polymorphisms using a random or fixed effect model based on the calculated heterogeneity between the studies [50]. The *χ*2-based *Q* statistics and the *I*^2^ statistics [51] were used to evaluate the between study heterogeneity, with *I*^2^ = 0% indicating no observed heterogeneity, 25% regarded as low, 50% as moderate, and 75% as high [52]. When *Q* test or the *I*^2^ test indicated significant heterogeneity between the studies (*p* < 0.10, *I*^2^ >50%), the random-effect model was used, otherwise, the fixed-effect model was applied. Additionally, Galbraith's plot was constructed to explore the weight each study had on the overall estimate and the contribution to the *Q* statistics for heterogeneity [53].

We also performed a one-way sensitivity analysis to check stability of the results. To assess the publication bias (where appropriate), we conducted Egger's asymmetry test (level of significance *p* < 0.05) [54]. All statistical analyses were performed using the STATA software package v.15 (STATA Corporation, College 162 Station, TX, USA), and statistical significance was set at *p* < 0.05.

## 3. Results

### 3.1. Search Results and Study Characteristics

**Figure 1:**
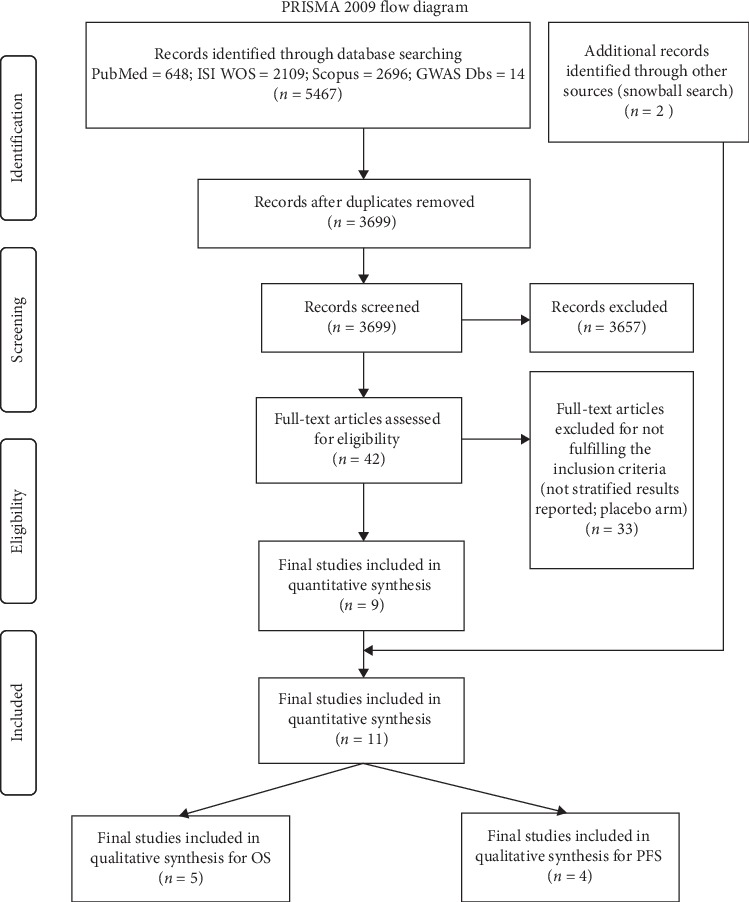
Flowchart depicting the literature search and study selection process.

Of 5467 records obtained through the screening of PubMed, ISI WOS, Scopus, and 14 GWAS databases, 3699 remained after removing the duplicates. After reading the titles and abstracts, 42 full text articles were assessed for the inclusion. We further excluded 33 papers for not fulfilling the inclusion criteria, leaving 9 studies as eligible. After inspection of references of the included studies, we additionally identified two studies, arriving to the 11 studies to be finally included in the review. Ultimately, 5 studies were incorporated in the quantitative synthesis for OS, and 4 were considered for PFS (Figure 1).

Our search results consisted of 10 cohort studies [25, 26, 31, 32, 55–60] and 1 randomized controlled trial (RCT) [61], conducted in high-income Western countries and in Asia. Overall quality of the included study was good, with two studies [56, 60] scoring maximum point on the NOS. Highest scores were demonstrated for most of the evaluated domains, while the domain of follow up adequacy was with the lowest score (Supplementary [Supplementary-material supplementary-material-1]). Sample size varied from 62 to 760 patients while median age from 55.2 to 67.0 years. Majority of patients were in clinical stages III and IV. Reported medium follow-up time ranged from 11.4 months up to 62.7 months, and TKIs used in the studies were gefitinib and erlotinib. Detailed description of the included studies is presented in Table 1.

All included studies investigated the OS and reported their findings for 10 different *EGFR* SNPs, namely, rs11543848, rs11568315, rs11977388, rs2075102, rs2227983, rs2293347, rs4947492, rs712829, rs712830, and rs7809028. The longest and the shortest median OS were reported for the homozygous wild genotype and a variant allele carriers for rs712829 (-216G>T), respectively [25]. Increased HR was observed in patients lacking wild-type (CA)16 allele (rs11568315) as compared with carriers of at least one (CA)16 allele [58]. On the other hand, lower HR (0.29; 95% CI: 0.10–0.83) indicating better prognosis was reported in homozygous carriers of less common g.106268G allele (rs4947492) [31], in carriers of at least one variant -216T (rs712829) allele (HR = 0.67; 95% CI: 0.48–0.94) [61], as well as in carriers of lower number of CA repeats (HR = 0.43; 95% CI: 0.23–0.78) [59], as compared with their corresponding genotypes. Interestingly, carriers of one or both variant alleles, as compared with homozygous wild genotype for 181946C>T (rs2293347), displayed increased HR according to one [31] and decreased HR according to other [32] investigated study. The details of OS, HR, and RR for each investigated SNP across the included studies are presented in Table 2.

Of all included studies, six [25, 26, 32, 56, 59, 61] reported PFS in relation to five different SNPs, with only one of them reporting the median PFS time [25]. On the other hand, TTP was reported in only two studies [55, 57] and were stratified according to four different SNPs (Table 3). Based on the PFS reports, better prognosis was associated with rs11568315, rs2293347, and rs712829 polymorphisms, i.e., with the presence of lower number of CA repeats [55] and variant 181946T [32] and -216T [56] alleles. In addition, lower number of CA repeats (rs11568315) was also associated with better TTP [55].

### 3.2. Quantitative Synthesis

**Figure 2:**
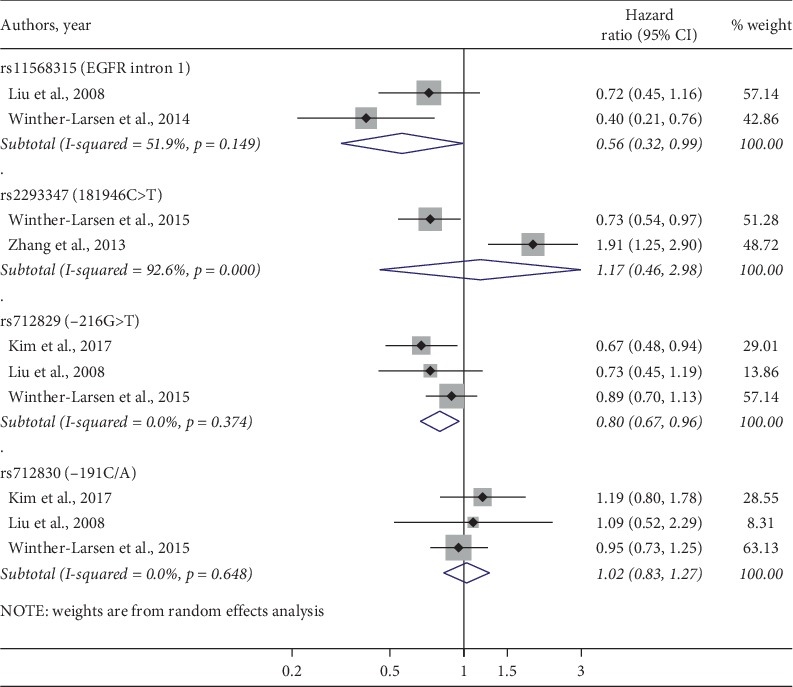
Forest plot reporting pooled HR and their 95% CI of four SNPs for the OS. The square size indicates the weight of each study and of pooled data.

Five studies [31, 32, 56, 59, 61] reported enough information about OS to be included in our meta-analysis, and the forest plot with pooled HR and their 95% CI of OS available for four SNPs, namely, rs11568315, rs712830, rs712829, and rs712830, is presented in Figure 2. Due to significant heterogeneity between the studies, the random effect model was applied. Egger test and Begg's correlation method demonstrated no evidence of publication bias. Our analysis revealed rs712829 (HR = 0.80, 95% CI: 0.67–0.96, *p*=0.01; heterogeneity *I*^2^ = 0%, *p*=0.37) and rs11568315 (HR = 0.56, 95% CI: 0.32–0.99, *p*=0.046; heterogeneity *I*^2^ = 51.9%, *p*=0.15) polymorphisms, more precisely the presence of at least one -216T variant allele and the presence of ≤16CA repeats, respectively, as the only positive prognostic factors for the OS, with no observed heterogeneity (Figure 2). The Egger test demonstrated no statistical evidence of publication bias for rs712829 and rs712830 (*p*=0.48 and *p*=0.6, respectively). As Galbraith's plot, performed to explore the potential sources of heterogeneity, identified the study of Winther-Larsen et al. [32], this study was omitted in one-way sensitivity analysis of rs712829. Yet, the overall HR remained significant and was only slightly changed to 0.69 (95% CI: 0.52–0.91, *p* < 0.008; heterogeneity *I*^2^ = 0%, *p*=0.78). Other investigated SNPs demonstrated increased pooled HR but without statistical significance.

**Figure 3:**
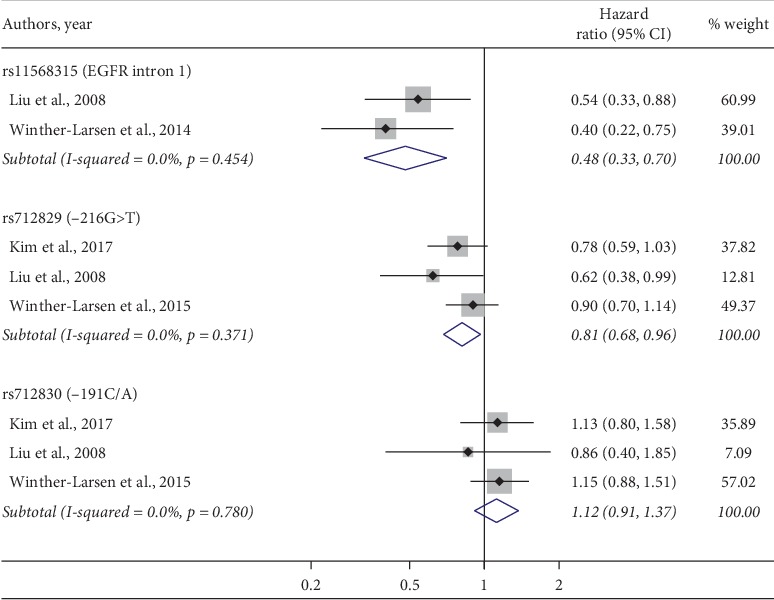
Forest plot reporting pooled HR and their 95% CI of three SNPs for the PFS. The square size indicates the weight of each study and of pooled data.

Four studies [32, 56, 59, 61] were included in the pooled analysis of PFS in patients stratified according to genotyping data available for three *EGFR* SNPs, i.e., rs11568315, rs712829, and rs712830. As there was no significant heterogeneity between the studies, the fixed effect model was applied. No significant publication bias was demonstrated by Egger tests (rs712829 and rs712830, *p*=0.19 and *p*=0.08, respectively) even though these tests for exploring the publication bias are underpowered with only few studies included. Again, the only significant factors, indicating better prognosis in NSCLC treated with TKIs, were the presence of at least one -216T variant allele (HR = 0.81, 95% CI: 0.68–0.96, *p*=0.02; heterogeneity *I*^2^ = 0%, *p*=0.37) and the presence of ≤16CA repeats (HR = 0.48, 95% CI: 0.33–0.7, *p* < 0.01; heterogeneity *I*^2^ = 0%, *p*=0.45) (Figure 3). The Galbraith plot identified the study of Winther-Larsen et al. [32], thus we omitted this study in the one-way sensitivity analysis for rs712829. The results confirmed statistical significance of rs712829-related HR, which was only slightly changed to 0.74 (95% CI: 0.58–0.94, *p*=0.01; heterogeneity *I*^2^ = 0%, *p*=0.42). The other investigated SNPs, namely, -191C>A (rs712830), displayed increased, albeit insignificant, pooled HR.

## 4. Discussion

The discovery of activating mutations in the *EGFR* gene from fifteen years ago represented a major breakthrough in the treatment of NSCLC [22, 23], as clinical responsiveness to TKIs, promising new treatment alternatives [17], turned out to be highly dependent on the presence of so-called “sensitizing” *EGFR* mutations [62]. Consequently, both of the first-generation TKIs, gefitinib and erlotinib, have been approved for the treatment of patients with metastatic NSCLC, but only if their tumors harbor *EGFR* exon 19 deletions or exon 21 (L858R) substitution mutations [63]. Nevertheless, neither *EGFR* mutation testing nor full TKI response is easy to achieve, as former postulates availability of samples of biopsied/resected tumor tissue or pleural effusion and appropriate methodology, expertise and equipment [64], while later is undermined by intrinsic or acquired resistance to TKI that exists or develops in majority of patients [24–26].

In overcoming these issues, numerous studies have been performed to disclose other important factors involved in response to TKI, aiming for those which could be more easily detected and also already present at the beginning of the treatment, hence useful as potential prediction markers for TKI-based therapy outcome. Significant research load has been focused on *EGFR* as the therapy target, revealing that certain germline variants of the *EGFR* gene could confer altered prognosis in their NSCLC-diagnosed carriers treated with TKI [31, 32, 55, 56, 58, 61]. However, the studies were either underpowered [25, 55, 56] or yielded conflicting results [26, 32, 58, 61], leaving the possibility of *EGFR* SNP-associated role in clinical responsiveness to TKIs insufficiently explored. To assess, consolidate, and integrate the available knowledge on this subject, we performed systematic review and meta-analysis of published reports on association between *EGFR* polymorphism and the survival of NSCLC patients. Of 10 *EFGR* SNPs evaluated in our study, four were reported to affect the response to TKI, namely, rs712829 (-216G>T) [56, 61], rs11568315 (CA repeat) [55, 56, 58, 59], rs2293347 (D994D) [31, 32], and rs4947492 [31]. However, pooled analysis of the available data revealed that only *EGFR* -216G>T and variable CA repeat polymorphisms significantly affect the prognosis of TKI-treated NSCLC patients, with longer OS and PFS associated with the presence of variant -216T allele and ≤16CA repeats.

The 5′-flanking region of the *EGFR* gene acts as a promoter by binding Sp1 transcription factor [65]. *EGFR*-216G>T SNP is located in one of the Sp1 binding sites, thus affecting initiation of the *EGFR* transcription [66]. Namely, it has been discovered that the replacement of G by T at this position increases the promoter activity and gene expression by 30% and 40%, respectively [67]. Furthermore, this effect proved to be unaffiliated to the presence of other polymorphisms in this region, as well as to the cell type or *EGFR* expression level [67]. The observation that the response on TKI only partly depends on the presence of *EGFR* activating mutations [68] opened the question of the yet-unexplained difference in therapy outcome, for which -216G>T polymorphism seemed like a reasonable answer. Therefore, most of the studies investigating the association between *EGFR* polymorphism and NSCLC TKI-based treatment included -216G>T. Many of them revealed that it significantly improves treatment outcome [25, 56, 69] and increases the risk of treatment-related toxicity [56, 57, 70]. However, some failed to observe such associations [32, 61, 69, 71], thus the overall conclusion regarding the importance of -216G>T has not been reached so far. In the present meta-analysis, whose advantage over previous publications lies in higher validity, reliability of the results [72], *EGFR* -216G>T was significantly associated with both OS and PFS in TKI-treated NSCLC patients. Our results therefore suggest the possibility that this *EGFR* polymorphism can be used as an easy-to-obtain and ever-present additional predictive factor in these patients, which could simplify the decision-making process during prescribing and improve the outcome of the therapy. It should be noted, however, that all the reports included in our study were based on either gefitinib or erlotinib treatment, thus our conclusions might not be necessarily relevant to the therapies based on newer TKIs, whose mechanism of action is slightly different [19, 73].

The first intron of *EGFR* has an important regulatory function, which relies on the presence of an enhancer element that stimulates promoter activity [74]. *EGFR* SNP rs11568315 is located close to enhancer in *EGFR* intron 1 and represents a variable simple sequence repeat (SSR) consisting of 14 up to 21 CA dinucleotides [75]. It has been observed that the transcription activity of *EGFR* declines with the increasing number of CA repeats, most probably due to alteration in DNA secondary structure, but also that this effect can be outweighed by other regulatory mechanisms [76]. To determine the possible role of this polymorphism in response to TKIs, numerous studies investigated NSCLC, but also other types of cancer whose therapy is *EGFR*-targeted. Some of the published reports conform in conclusion that the number of CA repeats affects the outcome of TKI-based therapy, with lower number of CA repeats corresponding to higher response rate [55, 60, 77, 78], longer time-to-progression [55, 56, 58, 59, 70], longer survival [58, 59, 77], and increased toxicity [79]. However, others did not detect any significant association [25, 26, 57, 61], deeming this *EGFR* polymorphism to be clinically unimportant. Out of eight CA repeat-related studies involved in our systematic review, in three the influence on survival has been reported, with carriers of 16 CA repeats (representing the shorter and the most frequent allele [80]) having longer OS [58, 59], and carriers of alleles shorter than or equal to 16 CA having longer PFS [56, 59], as compared with other NSCLC patients on gefitinib therapy. The present meta-analysis confirmed the observed effect of variable CA repeats on OS. The possible reasons of conflicting results in the literature might be the lack of consensus in regard to cutoff values defining shorter versus longer CA repeats [26, 81], the presence of linkage disequilibrium with other functional SNPs that remained undetected or unexplored [56, 66, 78], or interethnic differences in the allelic distribution [80]. Yet, our results indicate that the length of CA repeat in *EGFR* intron 1 could be used as another predictive marker for the outcome of TKI-based therapy in NSCLC patients.

The last two *EGFR* SNPs reported to affect the outcome of TKI-based treatment of NSCLC, namely, rs2293347 (D994D) and rs4947492, are currently the least explored. Both are localized within regulatory regions, as former resides in exon 25, i.e., within C-terminal domain [82], and later in the first intron of *EGFR* [83]. So far, the role of rs2293347 in the treatment of NSCLC patients has been investigated in three different studies [31, 32, 78], and all of them reported significant association of this polymorphism and the response to TKIs. Yet, while Ma et al. [78] and Zhang et al. [31] associated the presence of variant allele with shorter OS, shorter PFS, and lower response rate, Winther-Larsen et al. [32] reported the opposite, with variant (albeit major) allele carriers on gefitinib therapy exhibiting higher disease control rate and longer OS and PFS. This *EGFR* SNP is synonymous; hence, it does not lead to a change of the amino acid sequence. Nevertheless, it has been confirmed that even synonymous variations could alter protein amount, structure or function, by affecting mRNA stability, translational kinetics, and splicing [84]. Having in mind the localization of rs2293347, i.e., its proximity to TK domain [82], as well as the contradictory reports regarding its role in TKI efficacy and safety [31, 32, 78], this *EGFR* SNP could be considered a good candidate for future clinical trials. On the other hand, only one study of NSCLC treatment with TKIs evaluated the role of rs4947492 [31], reporting significant association of the variant allele with shorter OS [31]. This variation is believed to alter *EGFR* expression [31], yet linkage disequilibrium with other SNPs could also explain or affect its role in TKI-related treatment [83]. Anyhow, the observed effect would need further confirmation.

The present study harbors several limitations, including the lack or incompleteness of data regarding additional treatments used in included studies, which could affect the overall outcome of the therapy. Also, linkage disequilibrium that has been described among different *EGFR* SNPs, or between SNPs and *EGFR* activating mutations, was not always taken into account. In addition, only publically available reports were included in our study, thus the possibility of a publication bias cannot be completely excluded. Other types of bias that might affect included studies, e.g., selection bias and information bias, might be present too. Also, it would have been valuable to stratify our findings according to sociodemographic characteristics and/or environmental effect modifiers, but this was not feasible since the original datasets were not available to us. Finally, the number of available studies for most of the investigated SNPs was insufficient for any sound conclusion to be drawn. Nevertheless, our study has several advantages. We used comprehensive and rigorous methodology to obtain all available eligible studies. Quality of the included studies was rather high, confirmed by the appropriate quality measurement tools. Statistical power of our analyses was considerably increased in respect to any single study, because of a bigger number of cases that were pooled for different SNPs.

In conclusion, our study shows that out of ten investigated *EGFR* SNPs (rs11543848, rs11568315, rs11977388, rs2075102, rs2227983, rs2293347, rs4947492, rs712829, rs712830, and rs7809028), only four, namely, rs712829 (-216G>T), rs11568315 (CA repeat), rs2293347 (D994D) and rs4947492, have been reported to affect the outcome of TKI-based NSCLC treatment. Of these, only -216G>T and variable CA repeat polymorphisms have been confirmed by meta-analysis of available data to significantly affect OS and PFS in gefitinib- or erlotinib-treated NSCLC patients. To ascertain whether these SNPs affect the response to other TKIs, as well as whether other *EGFR* SNPs have a role in NSCLC treatment, additional studies are warranted.

## Figures and Tables

**Table 1:** General characteristics of the included studies.

Author, year	Country	Study period	No. of patients	Median age, years (range)	Gender (males, %)	Ethnicity (%)	Smokers (%)	Clinical stage (%)	Median follow-up in months	TKI (dose)	Additional therapy
Han et al., 2007 [55]	Korea	Jan 2002–Dec 2004	86	61 (30–87)	57.0	NR	55.8	IIIB (5.8); IV (94.2)	16.9	Gefitinib (250 mg/d)	NR

Ichihara et al., 2007 [26]	Japan	Nov 2000–May 2006	98	66 (NR)	63.0	NR	62.0	NR^b^	11.4^c^	Gefitinib (250 mg/d)	88.0% of patients previously treated with chemotherapy

Liu et al., 2008 [56]	USA and Canada	Dec 2000–Feb 2003	92	61 (36–87)	41.0	Caucasian (95); Asian (3); African American (2)	79.0	IIIB (7.0); IV (93.0)	28.5 (PFS); 29.9 (OS)	Gefitinib (NR)	85.0% of patients were previously, and 95% concurrently treated with chemotherapy

Giovannetti et al., 2010 [57]	Italy	NR	96	64 (NR)	57.3	NR	68.8	IIIB (9.4); IV (90.6)	NR	Gefitinib (250 mg/d)	84.5% of patients were previously treated with chemotherapy^d^

Tiseo et al., 2010 [58]	Italy	NR	91	67 (40–85)	61.5	Caucasian (100)	78.0	III (11.0); IV (89.0)	NR	Gefitinib (250 mg/d)	All patients were previously treated with chemotherapy

Nie et al., 2011 [60]	China	Jun 2002–Sep 2006–Jul 2010^a^	115^e^	57 (NR)	56.5	NR	NR	IV (83.5)	54.0	Gefitinib (250 mg/d) or erlotinib (150 mg/d)	All patients were previously treated with chemotherapy

Jung et al., 2012 [25]	Korea	Jan 2007–Dec 2010	71^f^	59 (34–85)	62.0	Asian (100)	57.7	NR	12.7	Gefitinib (250 mg/d) or erlotinib (150 mg/d)	All patients were previously treated with chemotherapy

Zhang et al., 2013 [31]	China	Jan 2008–Dec 2010	128	55 (32–80)	48.4	NR	32.0	IIIB (25.0); IV (75.0)	16.6	Gefitinib (250 mg/d)	All patients were previously treated with one or two other therapy options

Winther-Larsen et al., 2014 [59]	Denmark	Jan 2007–Oct 2011	62^g^	65 (33–88)	40.0	Caucasian (100)	16.0	IV (100)	52.2	Erlotinib (150 mg/d, dose reduced in case of side effects grade 2 or higher)	NR

Winther-Larsen et al., 2015 [32]	Denmark	Jan 2007–Apr 2014	331	64 (34–89)	46.0	Caucasian (100)	26.0	IV (100)	62.7	Erlotinib (150 mg/d, dose reduced in case of side effects grade 2 or higher)	84.0% and 10% of patients were previously treated with platinum-based and pemetrexed and/or docetacel-based chemotherapy, respectively

Kim et al., 2017 [61]	Italy and Canada	NR	760	62 (27–81)	66.3	East Asian (3.2); other (96.8)	79.3	IIIB (10.9); IV (89.1)	36.0	Erlotinib (150 mg/d)	50% of patients were treated with erlotinib, followed by cisplatin and gemcitabine at progression; other 50% were treated with cisplatin and gemcitabine, followed by erlotinib at progression

NR: not reported. ^a^Last follow-up. ^b^Based on the Eastern Cooperative Oncology Group performance status, there were 61.0% with grade 0/1 and 39.0% with grade 2/3/4. ^c^For survivors. ^d^Additional 127 chemotherapy-treated/gefitinib-nontreated NSCLC patients were used as a comparison. ^e^70 on gefitinib and 45 on erlotinib. ^f^37 on gefitinib and 34 on erlotinib. ^g^All were *EGFR* mutation positive.

**Table 2:** *EGFR* genotype and overall survival of NSCLC patients treated with TKIs.

dbSNP-ID	Variant type, location, and/or consequence	Author, year (ref)	Genotyping platform used	Genotype	No. of patients (%)	Median OS (95% CI) in months	HR (95% CI)	RR (95% CI)
rs11534848	Missense variant, 1562G>A, R521K	Zhang et al., 2013 [31]	MassARRAY system	AA	48 (35.5)	10.0 (4.2–15.8)	Reference	NA
				AG	66 (48.9)	16.8 (7.0–26.6)	1.53 (0.94–2.51)	NA
				GG	21 (15.6)	29.4 (4.9–53.9)	1.84 (0.86–3.95)	NA

rs11568315	Intron variant, g.55020560_55020561AC[n]	Giovannetti et al., 2010 [57]	TaqMan assay	Both alleles ≤ 16CA	30 (31.9)	7.9 (3.5–12.2)	NA	NA
				At least 1 allele > 16CA	64 (68.1)	11.6 (6.5–16.7)	NA	NA
		Nie et al., 2011 [60]	PCR-RFLP and sequencing	At least 1 allele ≤ 16CA	66 (57.4)	15.9 (9.4–22.4)	NA	Reference
				Both alleles > 16CA	49 (42.6)	10.7 (4.7–16.8)	NA	0.81 (0.55–1.19)
		Tiseo et al., 2010 [58]	Fluorescent PCR and capillary electrophoresis	At least 1 allele = 16CA	56 (74.0)	12 (9.0–15.0)	Reference	NA
				Both alleles ≠ 16CA	20 (26.0)	4 (1.0–8.0)	**1.95 (1.12–3.7)**	NA
		Ichihara et al., 2007 [26]	PCR and sequencing	Short allele ≥ 19CA or the sum of alleles ≥ 39CA	63 (64.3)	NA	Reference	NA
				Short allele < 19CA or the sum of alleles < 39CA	35 (35.7)	NA	0.96 (0.50–1.86)	NA
		Kim et al., 2017 [61]	TaqMan assay and sequencing	Both alleles ≤ 16CA	74 (28.0)	NA	Reference	NA
				At least 1 allele > 16CA	188 (72.0)	NA	0.89 (0.64–1.24)	NA
		Liu et al., 2008 [56]	PCR-RFLP	At least 1 allele > 16CA	59 (64.0)	NA	Reference	NA
				Both alleles ≤ 16CA	33 (36.0)	NA	0.72 (0.45–1.16)	NA
		Winther Larsen et al., 2014 [59]	PCR-RFLP and capillary electrophoresis	Any allele ≤ 16CA	44 (71.0)	19.6 (11.9–27.3)	**0.43 (0.23–0.78)**	NA
				Both alleles > 16CA	18 (29.0)	8.4 (5.0–11.9)	Reference	NA

rs11977388	Intron variant, g.150522T>C	Zhang et al., 2013 [31]	MassARRAY system	TT	50 (39.4)	13.2 (6.1–20.3)	Reference	NA
				TC	64 (50.4)	16.8 (7.2–26.4)	1.26 (0.80–2.00)	NA
				CC	13 (10.2)	16.5 (0.0–40.4)	1.34 (0.56–3.18)	NA

rs2075102	Intron variant, g.171581C>A	Zhang et al., 2013 [31]	MassARRAY system	CC	90 (70.3)	11.8 (5.9–17.7)	Reference	NA
				CA	33 (25.8)	16.8 (12.8–20.8)	0.91 (0.55–1.49)	NA
				AA	5 (3.9)	29.4 (NA)	1.15 (0.34–3.90)	NA

rs2227983	Missense variant, 1562G>A, R497K	Giovannetti et al., 2010 [57]	TaqMan assay	GG or GA	81 (88.0)	7.4 (6.5–8.4)	NA	NA
				AA	11 (12.0)	8.0 (0.0–17.3)	NA	NA
		Nie et al., 2011 [60]	PCR-RFLP and sequencing	AA	43 (37.4)	17.4 (3.4–31.5)	NA	Reference
				AG	47 (40.9)	14.3 (7.0–21.6)	NA	0.81 (0.49–1.34)
				GG	25 (21.7)	12.3 (1.1–23.4)	NA	0.71 (0.43–1.17)
		Liu et al., 2008 [56]	PCR-RFLP	AA	43 (47.0)	NA	Reference	NA
				GG or GA	49 (53.0)	NA	1.24 (0.74–1.95)	NA
rs2293347	Synonymous variant, 181946C>T, D994D	Zhang et al., 2013 [31]	MassARRAY system	GG	59 (46.1)	21.0 (14.0–27.9)	Reference	NA
				GA	59 (46.1)	15.0 (8.3–21.8)	**1.75 (1.08–2.86)**	NA
				AA	10 (7.8)	2.0 (0.0–5.4)	**2.44 (1.06–5.56)**	NA
		Winther-Larsen et al., 2015 [32]	AS-PCR or PCR followed by sequencing	CC	252 (80.0)	NA	Reference	NA
				CT or TT	64 (20.0)	NA	**0.73 (0.54–0.97)**	NA

rs4947492	Intron variant, g.106268G>A	Zhang et al., 2013 [31]	MassARRAY system	AA	55 (43.0)	14.9 (6.9–22.8)	Reference	NA
				AG	60 (46.9)	11.8 (3.6–20.0)	0.86 (0.53–1.39)	NA
				GG	13 (10.1)	24.6 (NA)	**0.29 (0.10–0.83)**	NA

rs712829	5′ UTR variant, g.5031G>T, -216G>T	Giovannetti et al., 2010 [57]	TaqMan assay	GG	34 (36.2)	8.0 (3.0–13.0)	NA	NA
				GT or TT	60 (63.8)	11.6 (5.7–17.5)	NA	NA
		Jung et al., 2012 [25]	PCR-RFLP or PCR followed by sequencing	GG	63 (88.7)	29.5 (17.4–41.7)	NA	NA
				GT	8 (11.3)	1.4 (3.7–39.5)	NA	NA
		Kim et al., 2017 [61]	Taqman PCR and sequencing	GG	78 (32.0)	NA	Reference	NA
				GT or TT	162 (68.0)	NA	**0.67 (0.48–0.94)**	NA
		Winther-Larsen et al., 2015 [32]	AS-PCR or PCR followed by sequencing	GG	134 (42.0)	NA	Reference	NA
				GT or TT	182 (58.0)	NA	0.89 (0.70–1.13)	NA
		Liu et al., 2008 [56]	PCR-RFLP	GG	34 (37.0)	NA	Reference	NA
				GT or TT	58 (63.0)	NA	0.73 (0.45–1.19)	NA

rs712830	5′ UTR variant, g.5056A>C, -191C/A	Giovannetti et al., 2010 [57]	TaqMan assay	CC	78 (83.0)	7.9 (7.0–8.7)	NA	NA
				CA or AA	16 (17.0)	6.0 (2.8–9.2)	NA	NA
		Kim et al., 2017 [61]	Taqman PCR and sequencing	CC	195 (81.0)	NA	Reference	NA
				CA or AA	45 (19.0)	NA	1.19 (0.80–1.78)	NA
		Winther-Larsen et al., 2015 [32]	AS-PCR or PCR followed by sequencing	CC	236 (75.0)	NA	Reference	NA
				CA or AA	80 (25.0)	NA	0.95 (0.73–1.25)	NA
		Liu et al., 2008 [56]	PCR-RFLP	CC	81 (88.0)	NA	Reference	NA
				CA or AA	11 (12.0)	NA	1.09 (0.52–2.29)	NA

rs7809028	Regulatory region variant, g.198953G>A	Zhang et al., 2013 [31]	MassARRAY system	GG	60 (49.2)	20.9 (7.4–34.4)	Reference	NA
				GA	53 (43.4)	16.5 (10.2–20.4)	0.74 (0.45–1.21)	NA
				AA	9 (7.4)	2.0 (1.0–3.1)	0.52 (0.22–1.21)	NA

OS: overall survival; HR: hazard ratio; RR: relative risk; NA: not available.

**Table 3:** *EGFR* genotype and survival (PFS and TTP) of NSCLC patients treated with TKIs.

dbSNP-ID	Variant type, location, and/or consequence	Author, year (ref)	Genotyping platform used	Genotype	No. of patients (%)	Progression-free survival (PFS) (95% CI)	Time-to-progression (TTP) (95% CI)
rs11568315	Intron variant, g.55020560_55020561AC[n]	Giovannetti et al., 2010 [57]	TaqMan assay	Both alleles ≤ 16CA	30 (31.9)	NA	3.2 (0.7–5.7)^b^
				At least 1 allele > 16CA	64 (68.1)	NA	3.1 (2.4–3.8)^b^
		Han et al., 2007 [55]	PCR and fragment length analysis	Both alleles ≥ 38CA	46 (53.5)	NA	Reference
				Both alleles ≤ 37CA	40 (46.5)	NA	**0.54 (0.34–0.88)** ^a^
		Ichihara et al., 2007 [26]	PCR and sequencing	Short allele ≥ 19CA or the sum of alleles ≥ 39CA	63 (64.3)	Reference	NA
				Short allele < 19CA or the sum of alleles < 39CA	35 (35.7)	1.08 (0.63–1.86)^a^	NA
		Kim et al., 2017 [61]	TaqMan assay and sequencing	Both alleles ≤ 16CA	74 (28.0)	Reference	NA
				At least 1 allele > 16CA	188 (72.0)	0.94 (0.71–1.25)^a^	NA
		Liu et al., 2008 [56]	PCR-RFLP	At least 1 allele > 16CA	59 (64.0)	Reference	NA
				Both alleles ≤ 16CA	33 (36.0)	**0.54 (0.33–0.88)^a^**	NA
		Winther Larsen et al., 2014 [59]	PCR-RFLP and capillary electrophoresis	Any allele ≤ 16CA	44 (71.0)	**0.39 (0.22–0.70)^a^**	NA
				Both alleles > 16CA	18 (29.0)	Reference	NA

rs2227983	Missense variant, 1562G>A, R497K	Giovannetti et al., 2010 [57]	TaqMan assay	GG or GA	81 (88.0)	NA	3.3 (2.4–5.0)^b^
				AA	11 (12.0)	NA	3.1 (1.5–4.7)^b^
		Liu et al., 2008 [56]	PCR-RFLP	AA	43 (47.0)	Reference	NA
				GG or GA	49 (53.0)	1.54 (0.98–2.42)^a^	NA

rs2293347	Synonimous variant, 181946C>T, D994D	Winther-Larsen et al., 2015 [32]	AS-PCR or PCR followed by sequencing	CC	252 (80.0)	Reference	NA
				CT or TT	64 (20.0)	**0.74 (0.55–0.99)^a^**	NA

rs712829	5′ UTR variant, g.5031G>T, -216G>T	Giovannetti et al., 2010 [57]	TaqMan assay	GG	34 (36.2)	NA	3.2 (2.6–3.8)^b^
				GT or TT	60 (63.8)	NA	3.2 (0.8–5.7)^b^
		Jung et al., 2012 [25]	PCR-RFLP or PCR followed by sequencing	GG	63 (88.7)	5.1 (2.7–7.5)^b^	NA
				GT	8 (11.3)	16.6 (5.8–27.5)^b^	NA
		Kim et al., 2017 [61]	Taqman PCR and sequencing	GG	78 (32.0)	Reference	NA
				GT or TT	162 (68.0)	0.78 (0.59–1.03)^a^	NA
		Winther-Larsen et al., 2015 [32]	AS-PCR or PCR followed by sequencing	GG	134 (42.0)	Reference	NA
				GT or TT	182 (58.0)	0.90 (0.70–1.14)^a^	NA
		Liu et al., 2008 [56]	PCR-RFLP	GG	34 (37.0)	Reference	NA
				GT or TT	58 (63.0)	**0.62 (0.38–0.99)^a^**	NA
rs712830	5′ UTR variant, g.5056A>C, -191C/A	Giovannetti et al., 2010 [57]	TaqMan assay	CC	78 (83.0)	NA	3.2 (2.5–3.9)^b^
				CA or AA	16 (17.0)	NA	3.2 (3.0–3.4)^b^
		Kim et al., 2017 [61]	Taqman PCR and sequencing	CC	195 (81.0)	Reference	NA
				CA or AA	45 (19.0)	1.13 (0.8–1.58)^a^	NA
		Winther-Larsen et al., 2015 [32]	AS-PCR or PCR followed by sequencing	CC	236 (75.0)	Reference	NA
				CA or AA	80 (25.0)	1.15 (0.88–1.51)^a^	NA
		Liu et al., 2008 [56]	PCR-RFLP	CC	81 (88.0)	Reference	NA
				CA or AA	11 (12.0)	0.86 (0.40–1.85)^a^	NA

^a^Hazard ratio (HR); ^b^Median (months); NA–not available; in bold, significant result at the level <0.05.

## Supplementary Materials

Supplementary Materials Supplementary Table 1: methodological quality of the included studies: based on A, the Newcastle-Ottawa Quality Assessment Scale for cohort studies, and on B, Jadad scale for RCT. Supplementary Figure 2: funnel plot for publication bias evaluation: overall survival for rs712829 (-216G>T). Supplementary Figure 3: funnel plot for publication bias evaluation: overall survival for rs712830 (-191C/A). Supplementary Figure 4: Galbraith plot for assessing heterogeneity: overall survival for rs712829 (-216G>T). Supplementary Figure 5: Galbraith plot for assessing heterogeneity: overall survival for rs712830 (-191C/A). Supplementary Figure 6: funnel plot for publication bias evaluation: progression-free survival for rs712829 (-216G>T). Supplementary Figure 7: funnel plot for publication bias evaluation: progression-free survival for rs712830 (-191C/A). Supplementary Figure 8: Galbraith plot for assessing heterogeneity: PFS for rs712829 (-216G>T). Supplementary Figure 8: Galbraith plot for assessing heterogeneity: PFS for rs712830 (-191C/A).

## References

[1] Lu T., Yang X., Huang Y. (2019). Trends in the incidence, treatment, and survival of patients with lung cancer in the last four decades. *Cancer Management and Research*.

[2] Lortet-Tieulent J., Renteria E., Sharp L. (2015). Convergence of decreasing male and increasing female incidence rates in major tobacco-related cancers in Europe in 1988–2010. *European Journal of Cancer*.

[3] Bray F., Ferlay J., Laversanne M. (2015). Cancer incidence in five continents: inclusion criteria, highlights from volume X and the global status of cancer registration. *International Journal of Cancer*.

[4] Ferlay J., Colombet M., Soerjomataram I. (2019). Estimating the global cancer incidence and mortality in 2018: GLOBOCAN sources and methods. *International Journal of Cancer*.

[5] Bray F., Ferlay J., Soerjomataram I., Siegel R. L., Torre L. A., Jemal A. (2018). Global cancer statistics 2018: GLOBOCAN estimates of incidence and mortality worldwide for 36 cancers in 185 countries. *CA: A Cancer Journal for Clinicians*.

[6] Hubbard M. O., Fu P., Margevicius S., Dowlati A., Linden P. A. (2012). Five-year survival does not equal cure in non-small cell lung cancer: a surveillance, epidemiology, and end results-based analysis of variables affecting 10- to 18-year survival. *The Journal of Thoracic and Cardiovascular Surgery*.

[7] Vijayvergia N., Shah P. C., Denlinger C. S. (2015). Survivorship in non-small cell lung cancer: challenges faced and steps forward. *Journal of the National Comprehensive Cancer Network*.

[8] Cubillos-Angulo J. M., Fukutani E. R., Cruz L. A. B. (2020). Systems biology analysis of publicly available transcriptomic data reveals a critical link between AKR1B10 gene expression, smoking and occurrence of lung cancer. *PLoS One*.

[9] Cancer Genome Atlas Research Network (2014). Comprehensive molecular profiling of lung adenocarcinoma. *Nature*.

[10] Kris M. G., Johnson B. E., Berry L. D. (2014). Using multiplexed assays of oncogenic drivers in lung cancers to select targeted drugs. *JAMA*.

[11] Marshall A. L., Christiani D. C. (2013). Genetic susceptibility to lung cancer-light at the end of the tunnel?. *Carcinogenesis*.

[12] Luo S. Y., Lam D. C. (2013). Oncogenic driver mutations in lung cancer. *Translational Respiratory Medicine*.

[13] Arteaga C. (2002). Overview of epidermal growth factor receptor biology and its role as a therapeutic target in human neoplasia. *Seminars in Oncology*.

[14] Normanno N., Bianco C., De Luca A. (2001). The role of EGF-related peptides in tumor growth. *Frontiers in Bioscience*.

[15] Kosaka T., Yatabe Y., Endoh H., Kuwano H., Takahashi T., Mitsudomi T. (2004). Mutations of the epidermal growth factor receptor gene in lung cancer: biological and clinical implications. *Cancer Research*.

[16] Arora A., Scholar E. M. (2005). Role of tyrosine kinase inhibitors in cancer therapy. *Journal of Pharmacology and Experimental Therapeutics*.

[17] Barker A. J., Gibson K. H., Grundy W. (2001). Studies leading to the identification of ZD1839 (IRESSA): an orally active, selective epidermal growth factor receptor tyrosine kinase inhibitor targeted to the treatment of cancer. *Bioorganic & Medicinal Chemistry Letters*.

[18] Kobayashi K., Hagiwara K. (2013). Epidermal growth factor receptor (EGFR) mutation and personalized therapy in advanced nonsmall cell lung cancer (NSCLC). *Targeted Oncology*.

[19] Takeda M., Nakagawa K. (2019). First- and second-generation EGFR-TKIs are all replaced to osimertinib in chemo-naive EGFR mutation-positive non-small cell lung cancer?. *International Journal of Molecular Sciences*.

[20] Maemondo M., Inoue A., Kobayashi K. (2010). Gefitinib or chemotherapy for non-small-cell lung cancer with mutated EGFR. *New England Journal of Medicine*.

[21] Mok T. S., Wu Y.-L., Thongprasert S. (2009). Gefitinib or carboplatin-paclitaxel in pulmonary adenocarcinoma. *New England Journal of Medicine*.

[22] Lynch T. J., Bell D. W., Sordella R. (2004). Activating mutations in the epidermal growth factor receptor underlying responsiveness of non-small-cell lung cancer to gefitinib. *New England Journal of Medicine*.

[23] Paez J. G., Janne P. A., Lee J. C. (2004). EGFR mutations in lung cancer: correlation with clinical response to gefitinib therapy. *Science*.

[24] Morgillo F., Fasano M., Ciardiello F. (2016). Mechanisms of resistance to EGFR-targeted drugs: lung cancer. *ESMO Open*.

[25] Jung M., Cho B. C., Lee C. H. (2012). EGFRPolymorphism as a predictor of clinical outcome in advanced lung cancer patients treated withEGFR-TKI. *Yonsei Medical Journal*.

[26] Ichihara S., Toyooka S., Fujiwara Y. (2007). The impact of epidermal growth factor receptor gene status on gefitinib-treated Japanese patients with non-small-cell lung cancer. *International Journal of Cancer*.

[27] Jurisic V., Obradovic J., Pavlovic S. (2018). Epidermal growth factor receptor gene in non-small-cell lung cancer: the importance of promoter polymorphism investigation. *Analytical Cellular Pathology*.

[28] National Center for Biotechnology Information (2019). *Database of Single Nucleotide Polymorphisms (dbSNP), National Centerfor Biotechnology Information*.

[29] Tate J. G., Bamford S., Jubb H. C. (2019). The catalogue of somatic mutations in cancer, COSMIC. *Nucleic Acids Research*.

[30] Keedy V. L., Temin S., Somerfield M. R. (2011). American Society of Clinical Oncology provisional clinical opinion: epidermal growth factor receptor (EGFR) Mutation testing for patients with advanced non-small-cell lung cancer considering first-line EGFR tyrosine kinase inhibitor therapy. *Journal of Clinical Oncology*.

[31] Zhang L., Yuan X., Chen Y., Du X.-J., Yu S., Yang M. (2013). Role of EGFR SNPs in survival of advanced lung adenocarcinoma patients treated with Gefitinib. *Gene*.

[32] Winther-Larsen A., Nissen P. H., Jakobsen K. R., Demuth C., Sorensen B. S., Meldgaard P. (2015). Genetic polymorphism in the epidermal growth factor receptor gene predicts outcome in advanced non-small cell lung cancer patients treated with erlotinib. *Lung Cancer*.

[33] Horgan A. M., Yang B., Azad A. K. (2011). Pharmacogenetic and germline prognostic markers of lung cancer. *Journal of Thoracic Oncology*.

[34] Moher D., Liberati A., Tetzlaff J., Altman D. G. (2010). Preferred reporting items for systematic reviews and meta-analyses: the PRISMA statement. *International Journal of Surgery*.

[35] National Center for Biotechnology Information (2019). https://www.ncbi.nlm.nih.gov/pubmed.

[36] Scopus Database, 2019, https://www.scopus.com

[37] Web of Science, 2019, https://clarivate.com/products/web-of-science/

[38] Genome-Wide Association Studies (GWAS) Database, 2019, https://www.gwascentral.org/

[39] Genetic Associations and Mechanisms in Oncology (GAME-ON), 2019, https://epi.grants.cancer.gov/gameon/publications.html

[40] Human Genome Epidemiology (HuGE) Navigator, 2019, https://phgkb.cdc.gov/PHGKB/startPagePubLit.action

[41] National Human Genome Research Institute (NHGRI GWAS Catalog), 2019, https://www.ebi.ac.uk/gwas/

[42] The Database of Genotypes and Phenotypes, (dbGaP), 2019, https://www.ncbi.nlm.nih.gov/gap

[43] The GWASdb, 2019, http://jjwanglab.org/gwasdb

[44] The Italian Genome-wide Database (IGDB), 2019, https://www.iigm.it/site/

[45] The GRASP: Genome-Wide Repository of Associations between SNPs and Phenotypes, 2019, https://grasp.nhlbi.nih.gov/Search.aspx 10.1093/nar/gku1202 PMC4383982 25428361

[46] Green S., Benedetti J., Crowley J. (1997). *Clinical Trials in Oncology*.

[47] Tang P. A., Bentzen S. M., Chen E. X., Siu L. L. (2007). Surrogate end points for median overall survival in metastatic colorectal cancer: literature-based analysis from 39 randomized controlled trials of first-line chemotherapy. *Journal of Clinical Oncology*.

[48] The Newcastle-Ottawa Scale Coding Manual, http://www.ohri.ca/programs/clinical_epidemiology/nos_manual.pdf

[49] Jadad A. R., Moore R. A., Carroll D. (1996). Assessing the quality of reports of randomized clinical trials: is blinding necessary?. *Controlled Clinical Trials*.

[50] DerSimonian R., Laird N. (1986). Meta-analysis in clinical trials. *Controlled Clinical Trials*.

[51] Deeks J., Altman D., Bradburn M., egger M., Davey Smith G., Altman D. (2001). Statistical methods for examining heterogeneity and combining results from several studies in meta-analysis. *Systematic Reviews in Health Care: Meta-Analysis in Context*.

[52] Higgins J. P. T., Thompson S. G., Deeks J. J. (2003). Measuring inconsistency in meta-analyses. *BMJ*.

[53] Galbraith R. F. (1988). A note on graphical presentation of estimated odds ratios from several clinical trials. *Statistics in Medicine*.

[54] Ioannidis J. P. A., Trikalinos T. A. (2007). The appropriateness of asymmetry tests for publication bias in meta-analyses: a large survey. *Canadian Medical Association Journal*.

[55] Han S.-W., Jeon Y. K., Lee K.-H. (2007). Intron 1 CA dinucleotide repeat polymorphism and mutations of epidermal growth factor receptor and gefitinib responsiveness in non-small-cell lung cancer. *Pharmacogenetics and Genomics*.

[56] Liu G., Gurubhagavatula S., Zhou W. (2008). Epidermal growth factor receptor polymorphisms and clinical outcomes in non-small-cell lung cancer patients treated with gefitinib. *The Pharmacogenomics Journal*.

[57] Giovannetti E., Zucali P. A., Peters G. J. (2010). Association of polymorphisms in AKT1 and EGFR with clinical outcome and toxicity in non-small cell lung cancer patients treated with gefitinib. *Molecular Cancer Therapeutics*.

[58] Tiseo M., Rossi G., Capelletti M. (2010). Predictors of gefitinib outcomes in advanced non-small cell lung cancer (NSCLC): study of a comprehensive panel of molecular markers. *Lung Cancer*.

[59] Winther Larsen A., Nissen P. H., Meldgaard P., Weber B., Sorensen B. S. (2014). EGFR CA repeat polymorphism predict clinical outcome in EGFR mutation positive NSCLC patients treated with erlotinib. *Lung Cancer*.

[60] Nie Q., Yang X.-N., An S.-J. (2011). CYP1A1^∗^2A polymorphism as a predictor of clinical outcome in advanced lung cancer patients treated with EGFR-TKI and its combined effects with EGFR intron 1 (CA)n polymorphism. *European Journal of Cancer*.

[61] Kim L., Saieg M., Di Maio M. (2017). Biomarker analysis of the phase 3 TORCH trial for first line erlotinib versus chemotherapy in advanced non-small cell lung cancer patients. *Oncotarget*.

[62] Juan O., Popat S. (2017). Treatment choice in epidermal growth factor receptor mutation-positive non-small cell lung carcinoma: latest evidence and clinical implications. *Therapeutic Advances in Medical Oncology*.

[63] U.S. Food and Drug Administration, 2019, http://www.fda.gov

[64] Ellison G., Zhu G., Moulis A., Dearden S., Speake G., McCormack R. (2013). EGFRmutation testing in lung cancer: a review of available methods and their use for analysis of tumour tissue and cytology samples. *Journal of Clinical Pathology*.

[65] Ishii S., Xu Y. H., Stratton R. H., Roe B. A., Merlino G. T., Pastan I. (1985). Characterization and sequence of the promoter region of the human epidermal growth factor receptor gene. *Proceedings of the National Academy of Sciences*.

[66] Johnson A. C., Ishii S., Jinno Y. (1988). Epidermal growth factor receptor gene promoter. Deletion analysis and identification of nuclear protein binding sites. *The Journal of Biological Chemistry*.

[67] Liu W., Innocenti F., Wu M. H. (2005). A functional common polymorphism in a Sp1 recognition site of the epidermal growth factor receptor gene promoter. *Cancer Research*.

[68] Pao W., Miller V., Zakowski M. (2004). EGF receptor gene mutations are common in lung cancers from “never smokers” and are associated with sensitivity of tumors to gefitinib and erlotinib. *Proceedings of the National Academy of Sciences*.

[69] Zhang X., Fan J., Li Y. (2016). Polymorphisms in epidermal growth factor receptor (EGFR) and AKT1 as possible predictors of clinical outcome in advanced non-small-cell lung cancer patients treated with EGFR tyrosine kinase inhibitors. *Tumor Biology*.

[70] Liu G., Cheng D., Ding K. (2012). Pharmacogenetic analysis of BR.21, a placebo-controlled randomized phase III clinical trial of erlotinib in advanced non-small cell lung cancer. *Journal of Thoracic Oncology*.

[71] Shitara M., Sasaki H., Yokota K. (2012). Polymorphisms in intron 1 of the EGFR gene in non-small cell lung cancer patients. *Experimental and Therapeutic Medicine*.

[72] Leucht S., Kissling W., Davis J. M. (2009). How to read and understand and use systematic reviews and meta-analyses. *Acta Psychiatrica Scandinavica*.

[73] Nan X., Xie C., Yu X. (2017). EGFR TKI as first-line treatment for patients with advanced EGFR mutation-positive non-small-cell lung cancer. *Oncotarget*.

[74] Maekawa T., Imamoto F., Merlino G. T. (1989). Cooperative function of two separate enhancers of the human epidermal growth factor receptor proto-oncogene. *The Journal of Biological Chemistry*.

[75] Chi D. D., Hing A. V., Helms C. (1992). Two chromosome 7 dinucleotide repeat polymorphisms at gene loci epidermal growth factor receptor (EGFR) and pro alpha 2 (I) collagen (COL1A2). *Human Molecular Genetics*.

[76] Gebhardt F., Zänker K. S., Brandt B. (1999). Modulation of epidermal growth factor receptor gene transcription by a polymorphic dinucleotide repeat in intron 1. *Journal of Biological Chemistry*.

[77] Nie Q., Wang Z., Zhang G. C. (2007). The epidermal growth factor receptor intron1 (CA) n microsatellite polymorphism is a potential predictor of treatment outcome in patients with advanced lung cancer treated with Gefitinib. *European Journal of Pharmacology*.

[78] Ma F., Sun T., Shi Y. (2009). Polymorphisms of EGFR predict clinical outcome in advanced non-small-cell lung cancer patients treated with Gefitinib. *Lung Cancer*.

[79] Amador M. L., Oppenheimer D., Perea S. (2004). An epidermal growth factor receptor intron 1 polymorphism mediates response to epidermal growth factor receptor inhibitors. *Cancer Research*.

[80] Liu W., Innocenti F., Chen P. (2003). Interethnic difference in the allelic distribution of human epidermal growth factor receptor intron 1 polymorphism. *Clinical Cancer Research*.

[81] Dubey S., Stephenson P., Levy D. E. (2006). EGFR dinucleotide repeat polymorphism as a prognostic indicator in non-small cell lung cancer. *Journal of Thoracic Oncology*.

[82] Mitsudomi T., Yatabe Y. (2010). Epidermal growth factor receptor in relation to tumor development: EGFR gene and cancer. *FEBS Journal*.

[83] Hou W. G., Ai W. B., Bai X. G. (2012). Genetic variation in the EGFR gene and the risk of glioma in a Chinese Han population. *PLoS One*.

[84] Kimchi-Sarfaty C., Oh J. M., Kim I.-W. (2007). A “silent” polymorphism in the MDR1 gene changes substrate specificity. *Science*.

