# Supplementary material for: EGFR Polymorphism and Survival of NSCLC Patients Treated with TKIs: A Systematic Review and Meta-Analysis
Source: J Oncol. 2020 Mar 18;2020:1973241. doi: 10.1155/2020/1973241 (PMC7104312; doi:10.1155/2020/1973241)
Supplement: Supplementary Materials — Supplementary Table 1: methodological quality of the included studies: based on A, the Newcastle-Ottawa Quality Assessment Scale for cohort studies, and on B, Jadad scale for RCT. Supplementary Figure 2: funnel plot for publication bias evaluation: overall survival for rs712829 (-216G>T). Supplementary Figure 3: funnel plot for publication bias evaluation: overall survival for rs712830 (-191C/A). Supplementary Figure 4: Galbraith plot for assessing heterogeneity: overall survival for rs712829 (-216G>T). Supplementary Figure 5: Galbraith plot for assessing heterogeneity: overall survival for rs712830 (-191C/A). Supplementary Figure 6: funnel plot for publication bias evaluation: progression-free survival for rs712829 (-216G>T). Supplementary Figure 7: funnel plot for publication bias evaluation: progression-free survival for rs712830 (-191C/A). Supplementary Figure 8: Galbraith plot for assessing heterogeneity: PFS for rs712829 (-216G>T). Supplementary Figure 8: Galbraith plot for assessing heterogeneity: PFS for rs712830 (-191C/A). [file 1973241.f1.docx]

**Supplementary table 1. Methodological quality of the included studies, based on A) the Newcastle-Ottawa Quality Assessment Scale for cohort studies), and on B) Jadad scale for RCT**

|  | | | **Cohort studies** | | | | | | | | | | **RCT** |
| --- | --- | --- | --- | --- | --- | --- | --- | --- | --- | --- | --- | --- | --- |
|  |  |  | **Han et al., 2007 [55]** | **Ichihara et al., 2007** **[26]** | **Liu et al., 2008 [56]** | **Giovannetti et al., 2010 [57]** | **Tiseo et al., 2010 [58]** | **Nie et al., 2011 [60]** | **Jung et al., 2012 [25]** | **Zhang et al., 2013 [31]** | **Winther-Larsen et al., 2014 [59]** | **Winther-Larsen et al., 2015**  **[32]** | **Kim et al., 2017 [61]** |
| **A** | **Selection** | Representativeness of the exposed cohort |  | * | * | * |  | * |  | * |  | * | NA |
|  |  | Selection of the non exposed cohort | * | * | * | * | * | * | * | * |  | * | NA |
|  |  | Ascertainment of exposure | * | * | * | * | * | * | * | * |  | * | NA |
|  |  | Outcome of interest not present at start | * | * | * | * | * | * | * | * |  | * | NA |
|  | **Comparability** | Comparability of cohorts on the basis of the design or analysis | ** | ** | ** | ** | ** | ** | ** | ** |  | * | NA |
|  | **Outcome** | Assessment of outcome | * | * | * | * | * | * | * | * |  | * | NA |
|  |  | Was follow up long enough for outcomes to occur? | * |  | * |  |  | * | * | * |  | * | NA |
|  |  | Adequacy of follow up of cohorts |  |  | * |  |  | * | * |  |  | * | NA |
|  | **Overall Quality Score** | | **7** | **7** | **9** | **7** | **6** | **9** | **8** | **8** |  | **8** |  |
| **B** | Was the study described as randomized? | | NA | NA | NA | NA | NA | NA | NA | NA |  | NA | +1 |
|  | Was the method of randomization described and appropriate to conceal allocation? | | NA | NA | NA | NA | NA | NA | NA | NA |  | NA | +1 |
|  | If described and inappropriate, describe: | | NA | NA | NA | NA | NA | NA | NA | NA |  | NA | 0 |
|  | Was the study described as double blinded? | | NA | NA | NA | NA | NA | NA | NA | NA |  | NA | 0 |
|  | Was the method of double-blinding described and appropriate to maintain a double-blinding? | | NA | NA | NA | NA | NA | NA | NA | NA |  | NA | 0 |
|  | Was the method of blinding inappropriate? | | NA | NA | NA | NA | NA | NA | NA | NA |  | NA | 0 |
|  | Was there a description of withdrawals and drop outs? | | NA | NA | NA | NA | NA | NA | NA | NA |  | NA | +1 |
|  | **FINAL SCORE (0 – 5)** | |  |  |  |  |  |  |  |  |  |  | **3** |

NA - not applicable

Overall survival (OS) analyses

Supplementary figure 2. Funnel plot for publication bias evaluation Overall survival for rs712829 (-216G>T)

Supplementary figure 3. Funnel plot for publication bias evaluation Overall survival for rs712830 (-191C/A)


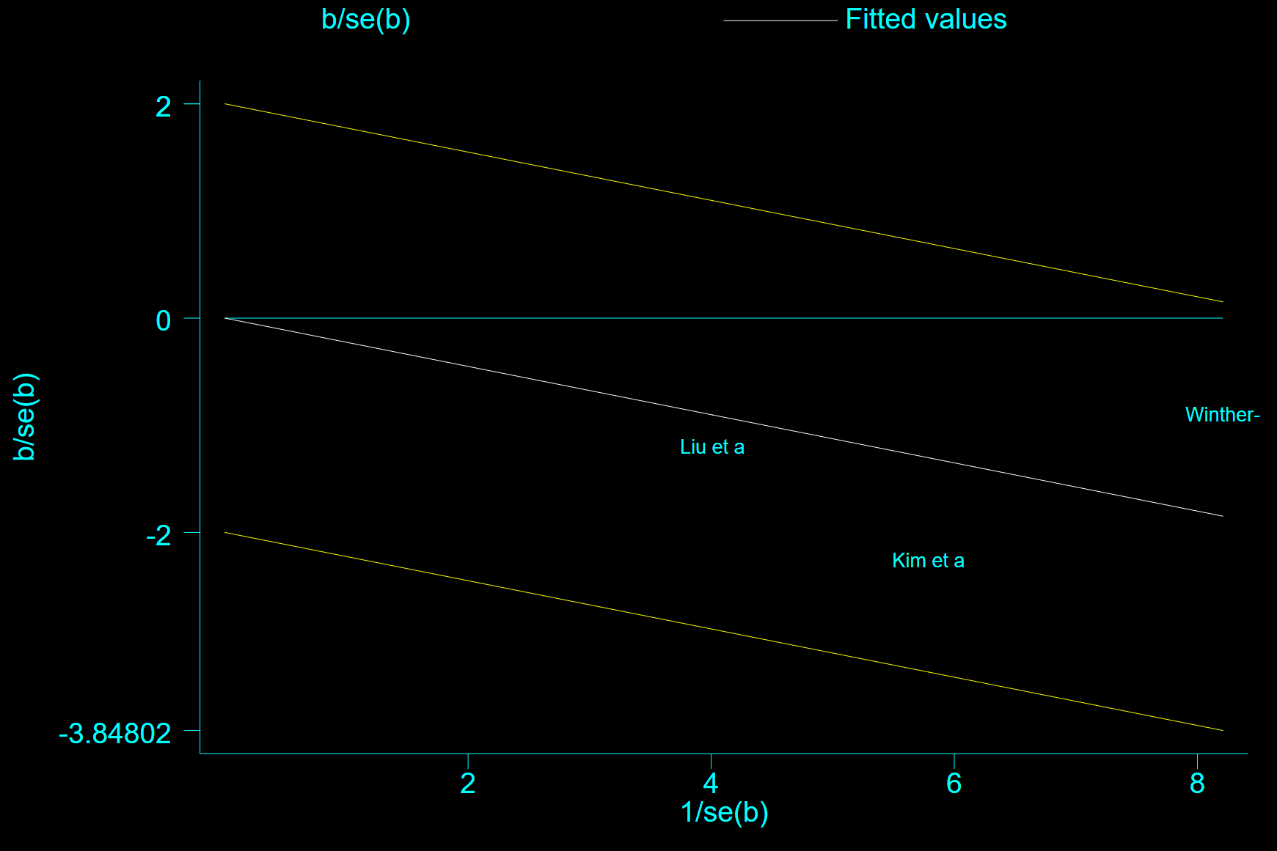


Supplementary figure 4. Galbraith plot for assessing heterogeneity - Overall survival for rs712829 (-216G>T)


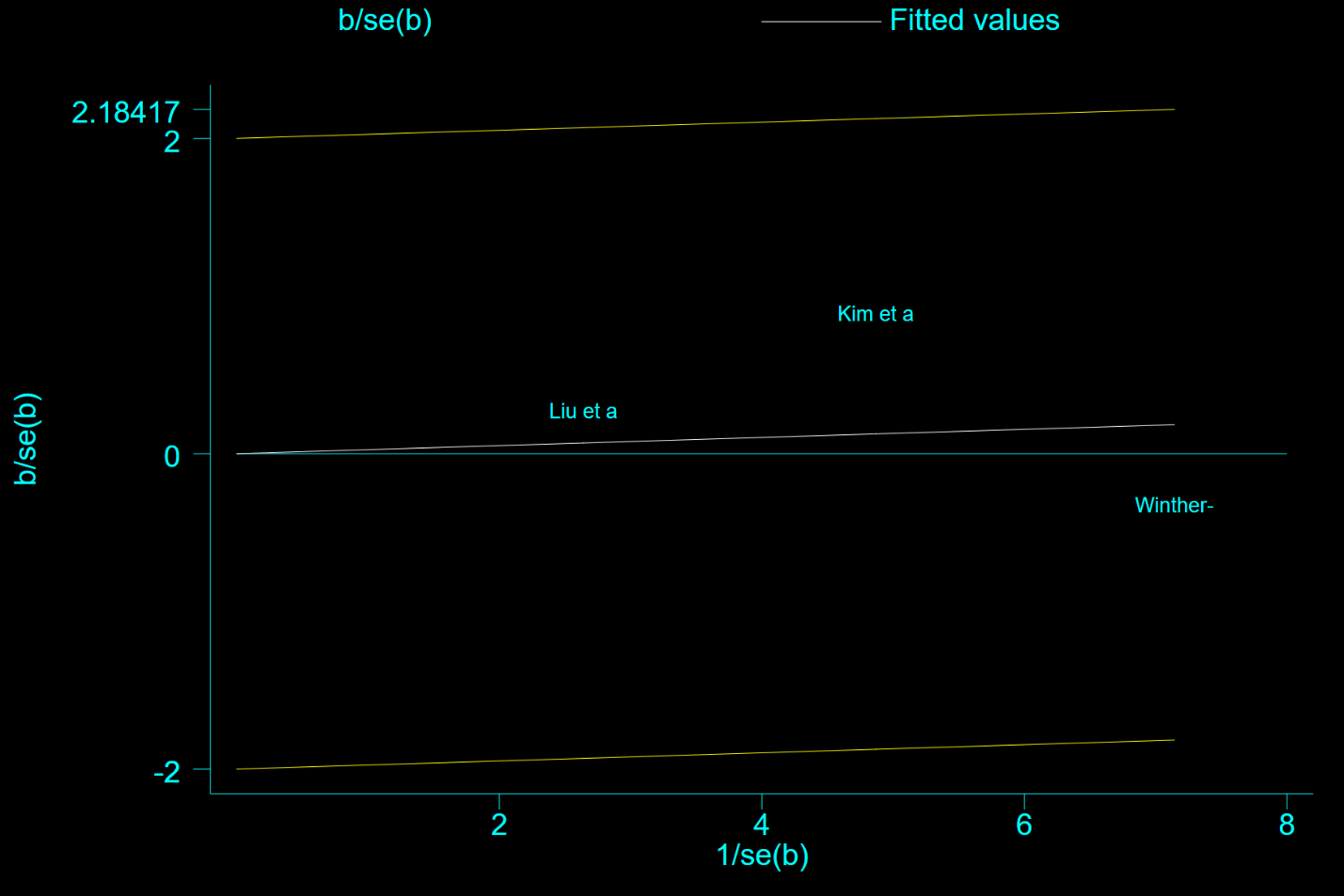


Supplementary figure 5. Galbraith plot for assessing heterogeneity - Overall survival for rs712830 (-191C/A)

Progression-free survival (PFS) analyses

Supplementary figure 6. Funnel plot for publication bias evaluation Progression-free survival for rs712829 (-216G>T)

Supplementary figure 7. Funnel plot for publication bias evaluation Progression-free survival for rs712830 (-191C/A)


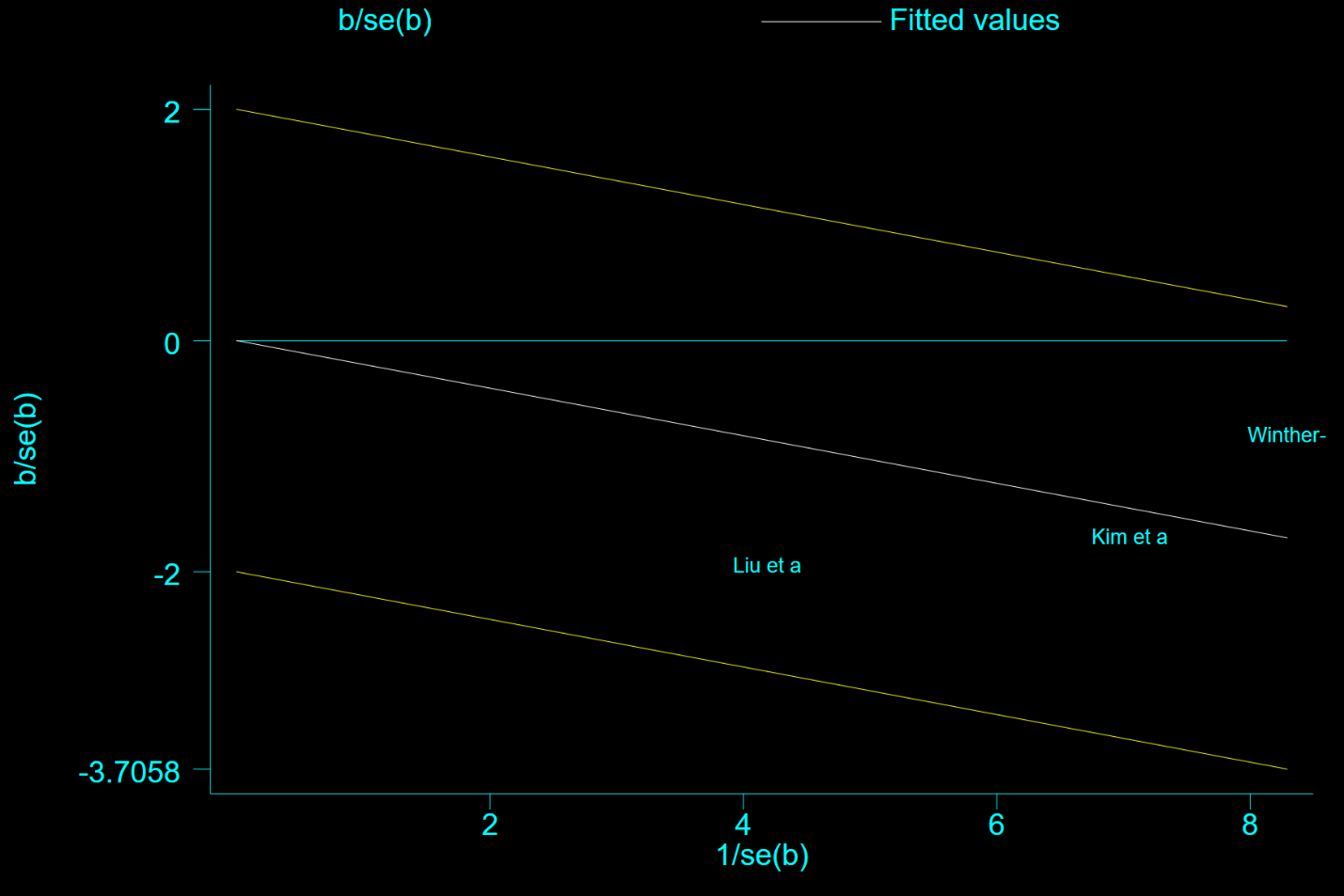


Supplementary figure 8. Galbraith plot for assessing heterogeneity - PFS for rs712829 (-216G>T)


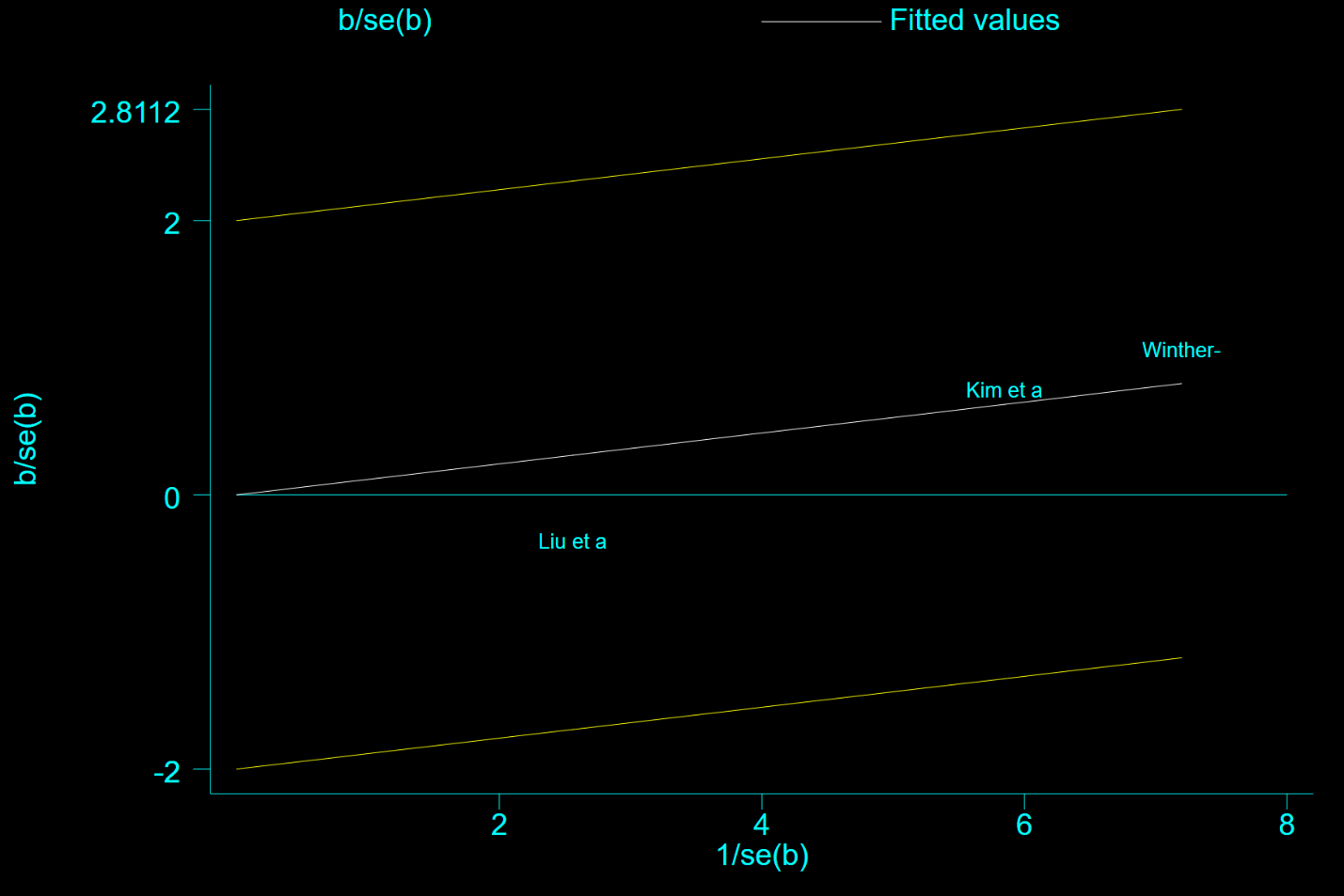


Supplementary figure 8. Galbraith plot for assessing heterogeneity - PFS for rs712830 (-191C/A)
